# 2-*C*-Phenyl­erythrono-1,4-lactone

**DOI:** 10.1107/S1600536809048478

**Published:** 2009-11-21

**Authors:** Tony V. Robinson, Dennis K. Taylor, Edward R. T. Tiekink

**Affiliations:** a Discipline of Chemistry, University of Adelaide, 5005 South Australia, Australia; b Discipline of Wine and Horticulture, University of Adelaide, Waite Campus, Glen, Osmond 5064, South Australia, Australia; c Department of Chemistry, University of Malaya, 50603 Kuala Lumpur, Malaysia

## Abstract

The title compound (systematic name: 3,4-dihydr­oxy-3-phenyl­furan-2-one), C_10_H_10_O_4_, features a five-membered γ-lactone ring with an envelope conformation at the C atom carrying the hydr­oxy group without the phenyl substituent. In the crystal, supra­molecular chains mediated by O—H⋯O hydrogen bonding are formed along the *a*-axis direction. These are consolidated in the crystal structure by C—H⋯O contacts.

## Related literature

For background on the leaf-closing substance of the tropical legume *Leucaena leucocephalam*, see: Ueda *et al.* (2001 ▶); Gogoi & Argade (2004 ▶); Koumbis *et al.* (2006 ▶). For the synthesis of polyhydroxy­ated compounds from 1,2-dioxines, see: Robinson *et al.* (2006 ▶, 2009 ▶); Valente *et al.* (2009 ▶); Pedersen *et al.* (2009 ▶).
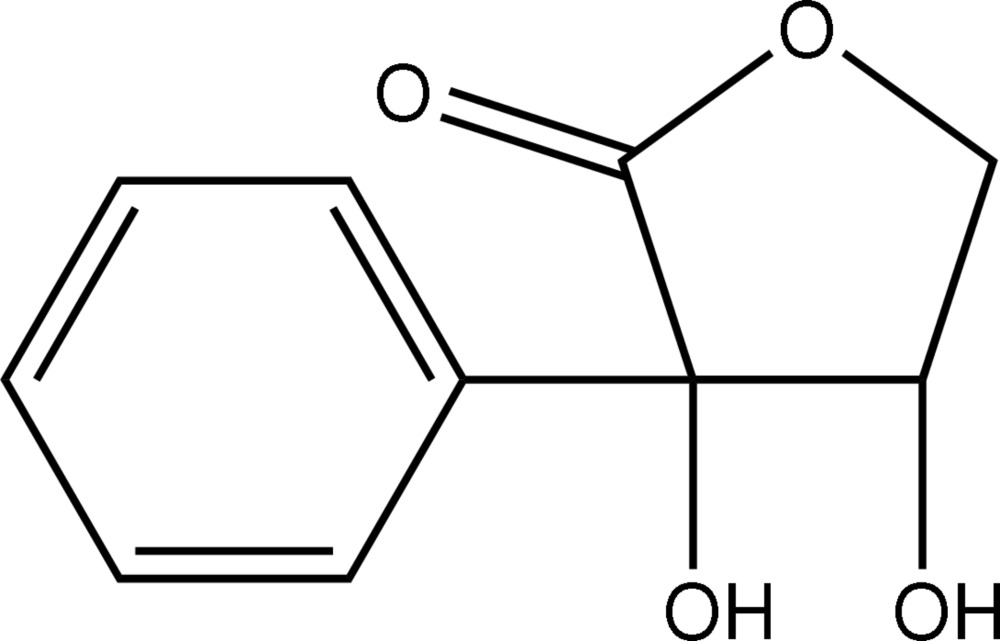


## Experimental

### 

#### Crystal data

C_10_H_10_O_4_
*M* *_r_* = 194.18
Monoclinic, 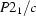

*a* = 6.485 (2) Å
*b* = 7.324 (3) Å
*c* = 18.962 (7) Å
β = 99.378 (7)°
*V* = 888.6 (5) Å^3^
*Z* = 4
Mo *K*α radiation
μ = 0.11 mm^−1^
*T* = 173 K
0.50 × 0.20 × 0.20 mm

#### Data collection

Rigaku AFC12κ/SATURN724 diffractometer
Absorption correction: multi-scan (*ABSCOR*; Higashi, 1995 ▶) *T*_min_ = 0.778, *T*_max_ = 1.000
21683 measured reflections
1834 independent reflections
1818 reflections with *I* > 2σ(*I*)
*R*_int_ = 0.028

#### Refinement

*R*[*F* ^2^ > 2σ(*F* ^2^)] = 0.040
*wR*(*F* ^2^) = 0.128
*S* = 1.21
1834 reflections
133 parameters
2 restraints
H-atom parameters constrained
Δρ_max_ = 0.28 e Å^−3^
Δρ_min_ = −0.27 e Å^−3^

### 

Data collection: *CrystalClear* (Rigaku/MSC, 2005 ▶); cell refinement: *CrystalClear*; data reduction: *CrystalClear*; program(s) used to solve structure: *SHELXS97* (Sheldrick, 2008 ▶); program(s) used to refine structure: *SHELXL97* (Sheldrick, 2008 ▶); molecular graphics: *ORTEPII* (Johnson, 1976 ▶) and *DIAMOND* (Brandenburg, 2006 ▶); software used to prepare material for publication: *publCIF* (Westrip, 2009 ▶).

## Supplementary Material

Crystal structure: contains datablocks global, I. DOI: 10.1107/S1600536809048478/sj2685sup1.cif

Structure factors: contains datablocks I. DOI: 10.1107/S1600536809048478/sj2685Isup2.hkl

Additional supplementary materials:  crystallographic information; 3D view; checkCIF report

## Figures and Tables

**Table 1:** Hydrogen-bond geometry (Å, °)

*D*—H⋯*A*	*D*—H	H⋯*A*	*D*⋯*A*	*D*—H⋯*A*
O3—H3o⋯O2^i^	0.84	1.95	2.7717 (19)	167
O4—H4o⋯O3^ii^	0.84	1.99	2.8188 (19)	168
C34—H34⋯O4^iii^	0.95	2.46	3.379 (2)	164

Symmetry codes: (i) 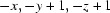; (ii) 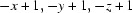; (iii) 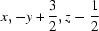.

**Fig. 1.**
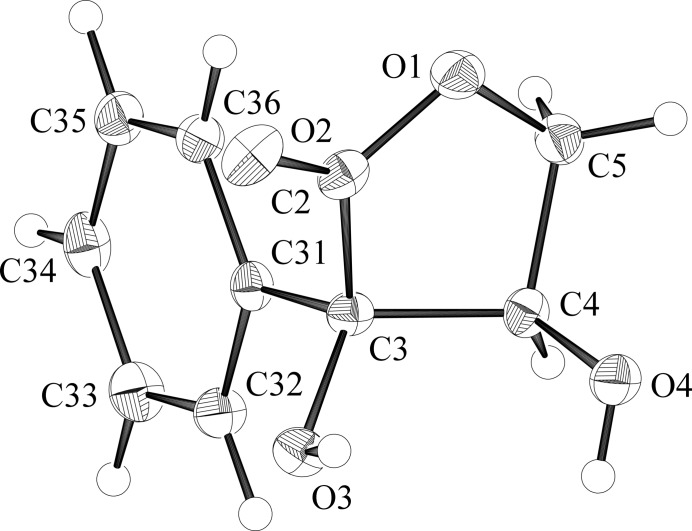
Molecular structure of (I) showing atom-labelling scheme and displacement ellipsoids at the 35% probability level.

**Fig. 2.**
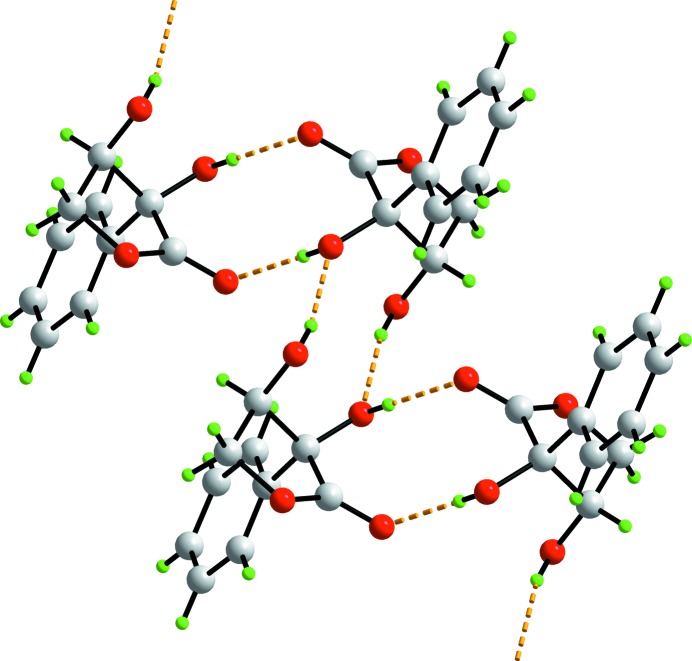
Supramolecular chain formation along the a axis in (I) mediated by O—H···O hydrogen bonds (orange dashed lines).

**Fig. 3.**
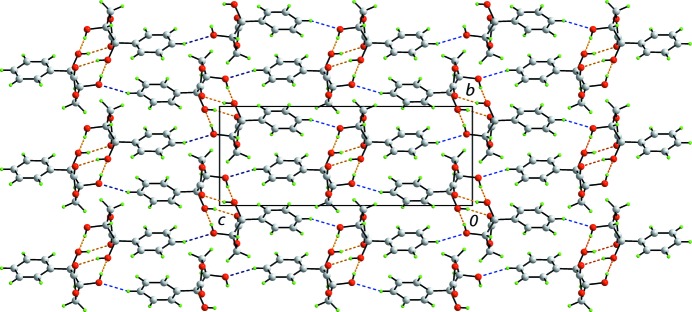
View in projection along the a axis in (I) showing the C—H···O contacts (blue dashed lines) linking the supramolecular chains (aligned along a) stabilized by O—H···O hydrogen bonds (orange dashed lines).

## supplementary crystallographic information

### Comment

Our recent investigations into the dihydroxyation of the alkene component of 1,2-dioxines has allowed access to a diverse range of polyhydroxyated compounds (Robinson *et al.*, 2006, 2009; Valente *et al.*, 2009). Application of this methodology to the synthesis of erythrono-γ-lactones, such as the title compound, (I), provided a concise route to potassium (2*R*,3*R*)-2,3,4-trihydroxy-2-methylbutanoate (Pedersen *et al.*, 2009), recently identified as a leaf-closing substance of the tropical legume Leucaena leucocephalam (Ueda *et al.*, 2001; Gogoi & Argade, 2004; Koumbis *et al.*, 2006).

The molecular structure of (I), Fig. 1, shows the five-membered γ-lactone ring to adopt an envelope conformation on the C4 atom, with this atom being orientated in the opposite direction to the phenyl ring. Both hydroxy substituents are orientated to the same side of the γ-lactone ring but the hydroxy-H atoms face opposite directions. This arrangement allows each molecule to bridge two neighbouring molecules *via O*—H_hydroxy_···O_hydroxy_ hydrogen bonds resulting in the formation of ten-membered {···HOC_2_O}_2_ synthons and the construction of supramolecular chains aligned along the *a* direction, Fig. 2 and Table 1. The chains are consolidated in the 3-D crystal structure *via* C—H···O contacts, Fig. 3 and Table 1.

### Experimental

For full synthetic procedures and characterization data see Pedersen *et al.* (2009). To a solution of 2,3-*O*-isopropylidene-2-*C*-phenyl-erythrono-1,4-lactone (159 mg, 0.68 mmol) in MeOH (10 ml) was added activated 50 W Dowex X8 resin (~ 1 g), and the mixture was stirred at 343 K until complete by TLC (~2–3 days). The reaction was allowed to cool and then filtered to remove the Dowex. The methanol was removed under reduced pressure and the residue was purified by flash chromatography to furnish (I) (115 mg, 87%) as a colourless solid. The pure material was recrystallized from a small amount of dichloromethane which was allowed to slowly evaporate at ambient temperature producing colourless prisms, m.pt. 381–382 K

### Refinement

Carbon-bound H-atoms were placed in calculated positions (C–H 0.95–1.00 Å) and were included in the refinement in the riding model approximation with *U*_iso_(H) set to 1.2–1.5*U*_eq_(C). The O–bound H-atoms were located in a difference Fourier map and refined with O–H restraints of 0.840±0.001 Å, and with *U_iso_*(H) = 1.5*U*_eq_(O).

### Crystal data

**Table:**

C_10_H_10_O_4_	*F*(000) = 408
*M**_r_* = 194.18	*D*_x_ = 1.452 Mg m^−^^3^
Monoclinic, *P*2_1_/*c*	Mo *K*α radiation, λ = 0.71070 Å
Hall symbol: -P 2ybc	Cell parameters from 3641 reflections
*a* = 6.485 (2) Å	θ = 2.2–27.5°
*b* = 7.324 (3) Å	µ = 0.11 mm^−^^1^
*c* = 18.962 (7) Å	*T* = 173 K
β = 99.378 (7)°	Block, colourless
*V* = 888.6 (5) Å^3^	0.50 × 0.20 × 0.20 mm
*Z* = 4	

### Data collection

**Table:**

Rigaku AFC12κ/SATURN724 diffractometer	1834 independent reflections
Radiation source: fine-focus sealed tube	1818 reflections with *I* > 2σ(*I*)
graphite	*R*_int_ = 0.028
ω scans	θ_max_ = 26.5°, θ_min_ = 2.2°
Absorption correction: multi-scan (*ABSCOR*; Higashi, 1995)	*h* = −8→7
*T*_min_ = 0.778, *T*_max_ = 1.000	*k* = −9→9
21683 measured reflections	*l* = −23→23

### Refinement

**Table:**

Refinement on *F*^2^	Primary atom site location: structure-invariant direct methods
Least-squares matrix: full	Secondary atom site location: difference Fourier map
*R*[*F*^2^ > 2σ(*F*^2^)] = 0.040	Hydrogen site location: inferred from neighbouring sites
*wR*(*F*^2^) = 0.128	H-atom parameters constrained
*S* = 1.21	*w* = 1/[σ^2^(*F*_o_^2^) + (0.0689*P*)^2^ + 0.2568*P*] where *P* = (*F*_o_^2^ + 2*F*_c_^2^)/3
1834 reflections	(Δ/σ)_max_ < 0.001
133 parameters	Δρ_max_ = 0.28 e Å^−^^3^
2 restraints	Δρ_min_ = −0.27 e Å^−^^3^

### Special details

**Table:**

Geometry. All e.s.d.'s (except the e.s.d. in the dihedral angle between two l.s. planes) are estimated using the full covariance matrix. The cell e.s.d.'s are taken into account individually in the estimation of e.s.d.'s in distances, angles and torsion angles; correlations between e.s.d.'s in cell parameters are only used when they are defined by crystal symmetry. An approximate (isotropic) treatment of cell e.s.d.'s is used for estimating e.s.d.'s involving l.s. planes.
Refinement. Refinement of *F*^2^ against ALL reflections. The weighted *R*-factor *wR* and goodness of fit *S* are based on *F*^2^, conventional *R*-factors *R* are based on *F*, with *F* set to zero for negative *F*^2^. The threshold expression of *F*^2^ > σ(*F*^2^) is used only for calculating *R*-factors(gt) *etc*. and is not relevant to the choice of reflections for refinement. *R*-factors based on *F*^2^ are statistically about twice as large as those based on *F*, and *R*- factors based on ALL data will be even larger.

### Fractional atomic coordinates and isotropic or equivalent isotropic displacement parameters (Å^2^)

**Table:**

	*x*	*y*	*z*	*U*_iso_*/*U*_eq_	
O1	−0.00052 (16)	0.88359 (15)	0.43151 (5)	0.0315 (3)	
O2	−0.14194 (16)	0.60605 (17)	0.42959 (6)	0.0353 (3)	
O3	0.27440 (16)	0.46977 (14)	0.44174 (5)	0.0274 (3)	
H3O	0.2312	0.4653	0.4811	0.041*	
O4	0.39267 (17)	0.77862 (15)	0.52318 (5)	0.0299 (3)	
H4O	0.4778	0.6920	0.5321	0.045*	
C2	0.0052 (2)	0.7033 (2)	0.42422 (7)	0.0260 (3)	
C3	0.2212 (2)	0.64295 (18)	0.41109 (7)	0.0224 (3)	
C4	0.3562 (2)	0.80116 (19)	0.44799 (7)	0.0242 (3)	
H4	0.4886	0.8182	0.4282	0.029*	
C5	0.2076 (2)	0.9607 (2)	0.43117 (8)	0.0292 (3)	
H5A	0.2386	1.0574	0.4678	0.035*	
H5B	0.2183	1.0132	0.3838	0.035*	
C31	0.2337 (2)	0.63650 (18)	0.33154 (7)	0.0229 (3)	
C32	0.4037 (2)	0.5486 (2)	0.31027 (8)	0.0287 (3)	
H32	0.5047	0.4900	0.3448	0.034*	
C33	0.4263 (3)	0.5462 (2)	0.23878 (8)	0.0336 (4)	
H33	0.5412	0.4839	0.2245	0.040*	
C34	0.2823 (3)	0.6341 (2)	0.18799 (8)	0.0323 (4)	
H34	0.2993	0.6336	0.1392	0.039*	
C35	0.1141 (3)	0.7223 (2)	0.20881 (8)	0.0313 (4)	
H35	0.0155	0.7833	0.1742	0.038*	
C36	0.0879 (2)	0.72227 (19)	0.28042 (8)	0.0272 (3)	
H36	−0.0299	0.7811	0.2942	0.033*	

### Atomic displacement parameters (Å^2^)

**Table:**

	*U*^11^	*U*^22^	*U*^33^	*U*^12^	*U*^13^	*U*^23^
O1	0.0283 (6)	0.0361 (6)	0.0304 (6)	0.0074 (4)	0.0055 (4)	−0.0040 (4)
O2	0.0265 (6)	0.0529 (7)	0.0274 (5)	−0.0055 (5)	0.0068 (4)	0.0059 (5)
O3	0.0346 (6)	0.0268 (5)	0.0221 (5)	0.0030 (4)	0.0078 (4)	0.0050 (4)
O4	0.0351 (6)	0.0355 (6)	0.0185 (5)	0.0059 (4)	0.0027 (4)	−0.0025 (4)
C2	0.0261 (7)	0.0363 (8)	0.0158 (6)	0.0018 (5)	0.0039 (5)	0.0016 (5)
C3	0.0239 (7)	0.0248 (7)	0.0189 (6)	0.0020 (5)	0.0043 (5)	0.0020 (5)
C4	0.0263 (7)	0.0280 (7)	0.0187 (6)	−0.0006 (5)	0.0048 (5)	−0.0012 (5)
C5	0.0326 (8)	0.0270 (7)	0.0277 (7)	0.0011 (6)	0.0040 (6)	−0.0018 (5)
C31	0.0267 (7)	0.0230 (6)	0.0192 (6)	−0.0029 (5)	0.0048 (5)	−0.0007 (5)
C32	0.0299 (7)	0.0326 (7)	0.0242 (7)	0.0030 (6)	0.0060 (5)	0.0000 (5)
C33	0.0372 (8)	0.0382 (8)	0.0281 (7)	0.0002 (6)	0.0131 (6)	−0.0041 (6)
C34	0.0471 (9)	0.0314 (7)	0.0199 (6)	−0.0079 (6)	0.0100 (6)	−0.0030 (5)
C35	0.0417 (9)	0.0281 (7)	0.0218 (7)	−0.0011 (6)	−0.0015 (6)	0.0024 (5)
C36	0.0302 (7)	0.0273 (7)	0.0236 (7)	0.0019 (5)	0.0027 (5)	−0.0007 (5)

### Geometric parameters (Å, °)

**Table:**

O1—C2	1.3286 (19)	C5—H5B	0.9900
O1—C5	1.4639 (19)	C31—C36	1.389 (2)
O2—C2	1.2085 (18)	C31—C32	1.392 (2)
O3—C3	1.4142 (16)	C32—C33	1.387 (2)
O3—H3O	0.8400	C32—H32	0.9500
O4—C4	1.4164 (16)	C33—C34	1.386 (2)
O4—H4O	0.8401	C33—H33	0.9500
C2—C3	1.5275 (19)	C34—C35	1.379 (2)
C3—C31	1.5243 (18)	C34—H34	0.9500
C3—C4	1.5486 (19)	C35—C36	1.396 (2)
C4—C5	1.515 (2)	C35—H35	0.9500
C4—H4	1.0000	C36—H36	0.9500
C5—H5A	0.9900		
			
C2—O1—C5	109.94 (11)	O1—C5—H5B	110.8
C3—O3—H3O	107.8	C4—C5—H5B	110.8
C4—O4—H4O	106.6	H5A—C5—H5B	108.8
O2—C2—O1	122.75 (13)	C36—C31—C32	119.22 (13)
O2—C2—C3	126.94 (14)	C36—C31—C3	122.46 (12)
O1—C2—C3	110.28 (12)	C32—C31—C3	118.24 (12)
O3—C3—C31	109.28 (10)	C33—C32—C31	120.24 (14)
O3—C3—C2	111.18 (11)	C33—C32—H32	119.9
C31—C3—C2	111.60 (10)	C31—C32—H32	119.9
O3—C3—C4	113.78 (11)	C34—C33—C32	120.46 (14)
C31—C3—C4	110.68 (10)	C34—C33—H33	119.8
C2—C3—C4	100.11 (11)	C32—C33—H33	119.8
O4—C4—C5	107.35 (11)	C35—C34—C33	119.55 (13)
O4—C4—C3	110.92 (11)	C35—C34—H34	120.2
C5—C4—C3	100.87 (11)	C33—C34—H34	120.2
O4—C4—H4	112.3	C34—C35—C36	120.39 (14)
C5—C4—H4	112.3	C34—C35—H35	119.8
C3—C4—H4	112.3	C36—C35—H35	119.8
O1—C5—C4	104.89 (11)	C31—C36—C35	120.14 (14)
O1—C5—H5A	110.8	C31—C36—H36	119.9
C4—C5—H5A	110.8	C35—C36—H36	119.9
			
C5—O1—C2—O2	172.88 (12)	C3—C4—C5—O1	33.83 (13)
C5—O1—C2—C3	−5.30 (14)	O3—C3—C31—C36	139.75 (13)
O2—C2—C3—O3	−31.23 (18)	C2—C3—C31—C36	16.37 (18)
O1—C2—C3—O3	146.85 (11)	C4—C3—C31—C36	−94.19 (15)
O2—C2—C3—C31	91.06 (16)	O3—C3—C31—C32	−43.46 (16)
O1—C2—C3—C31	−90.86 (13)	C2—C3—C31—C32	−166.83 (12)
O2—C2—C3—C4	−151.79 (13)	C4—C3—C31—C32	82.61 (15)
O1—C2—C3—C4	26.29 (13)	C36—C31—C32—C33	−0.3 (2)
O3—C3—C4—O4	−40.31 (15)	C3—C31—C32—C33	−177.20 (13)
C31—C3—C4—O4	−163.81 (11)	C31—C32—C33—C34	1.2 (2)
C2—C3—C4—O4	78.35 (12)	C32—C33—C34—C35	−0.9 (2)
O3—C3—C4—C5	−153.79 (11)	C33—C34—C35—C36	−0.4 (2)
C31—C3—C4—C5	82.71 (13)	C32—C31—C36—C35	−1.0 (2)
C2—C3—C4—C5	−35.13 (12)	C3—C31—C36—C35	175.79 (12)
C2—O1—C5—C4	−18.85 (14)	C34—C35—C36—C31	1.3 (2)
O4—C4—C5—O1	−82.33 (13)		

### Hydrogen-bond geometry (Å, °)

**Table:**

*D*—H···*A*	*D*—H	H···*A*	*D*···*A*	*D*—H···*A*
O3—H3o···O2^i^	0.84	1.95	2.7717 (19)	167
O4—H4o···O3^ii^	0.84	1.99	2.8188 (19)	168
C34—H34···O4^iii^	0.95	2.46	3.379 (2)	164

Symmetry codes: (i) −*x*, −*y*+1, −*z*+1; (ii) −*x*+1, −*y*+1, −*z*+1; (iii) *x*, −*y*+3/2, *z*−1/2.
